# Retraction: Downregulation of uPAR and Cathepsin B Induces Apoptosis via Regulation of Bcl-2 and Bax and Inhibition of the PI3K/Akt Pathway in Gliomas

**DOI:** 10.1371/journal.pone.0228688

**Published:** 2020-01-29

**Authors:** The PLOS ONE Editors

In a previous correction to this article [1, 2], an updated version of Fig 6 was provided to address image duplications involving the Control uPar data in Figs 6C and 6D, and the BAX blots shown in Figs 6E and 6F. In the updated figure, the panels of concern in Figs 6D and 6F were replaced with different data.

Additional concerns have since been noted for other figures [1]:

- In Fig 1D, similarities were noted between the Con and SV plots for 5310 cells.
- In Fig 1F, similarities were noted between the following panels: U251 TUNEL for pU and pC; U251 DAPI for pU and pC; 5310 DAPI for pU and pC.
- In the U251 BAX blot in Fig 2E, when levels are adjusted it appears as though the background in the SV lane differs from the background in other lanes.
- GAPDH data appear similar in Fig 4B, lanes 1–3, versus Fig 4C; and in Fig 4B, lanes 3–5 versus Fig 4D. These figure panels represent different experimental results.
- In Fig 4E, when levels are adjusted there appears to be a vertical discontinuity in the p-PI3K panel before the lane 2 band.

It is difficult to confirm the integrity of blot and gel images reported in the article’s figures in light of the poor image quality, and the uniform appearance of the background in these panels raises concerns.

The University of Illinois at Chicago investigated this work and found evidence of data falsification involving Figs 6C–6F.

In light of the above concerns that call into question the validity and reliability of the reported results and following the outcome of the institution’s investigation, the *PLOS ONE* Editors retract this article.

The authors either did not reply to the retraction notification or could not be reached.
